# Utility of Aprepitant in the Management of Pediatric Patients with Cyclical Vomiting Syndrome

**DOI:** 10.3390/medicines11080021

**Published:** 2024-12-11

**Authors:** Aravind Thavamani, Sindhoosha Malay, Jasmine Khatana, Sujithra Velayuthan, Senthilkumar Sankararaman

**Affiliations:** 1 Division of Pediatric Gastroenterology, Hepatology and Nutrition, UH Rainbow Babies and Children’s Hospital, Case Western Reserve University School of Medicine, 2101 Adelbert Rd, Cleveland, OH 44106, USA; aravind.thavamani@uhhospitals.org (A.T.); sujithrasen@gmail.com (S.V.); 2 Department of Pediatrics, UH Rainbow Babies and Children’s Hospital, Case Western Reserve University School of Medicine, 2101 Adelbert Rd, Cleveland, OH 44106, USA; sindhoosha.malay@uhhospitals.org; 3 Department of Pediatrics, Metro Health Medical Center, Case Western Reserve University School of Medicine, 2500 Metrohealth Dr, Cleveland, OH 44109, USA; jasminekhatana@gmail.com

**Keywords:** aprepitant, cyclic vomiting syndrome, population based, observational study, case control study, disorder of brain–gut interaction

## Abstract

**Introduction**: Cyclical vomiting syndrome (CVS) is a recurrent debilitating illness characterized by intense episodes of nausea and emesis with widely varied pharmacological management across the country. Aprepitant is now increasingly used in patients with CVS. The impact of aprepitant as an abortive therapy in the readmission of pediatric patients with CVS is currently unknown. **Methodology**: We analyzed all pediatric patients with a primary diagnosis of CVS using the ICD-10 code in the Pediatric Health Information System database of the Children’s Hospital Association. We evaluated the demographic data, comorbid conditions, and management details during the inpatient stay. CVS patients who received aprepitant during their inpatient hospitalization were compared with patients without aprepitant use. Seven-day readmission rate for CVS was used as the outcome variable to assess the effectiveness of the aprepitant in aborting an episode. Propensity score matching was used to match the two cohorts. **Results**: We analyzed 1775 patients of which 96 received aprepitant during the inpatient hospitalization. The aprepitant group had a more severe hospitalization course as evidenced by an increased duration of hospital stay (5 vs. 3 days) and total hospitalization costs ($11,790 vs. $6380). There were no significant differences in the 7-day (17% vs. 16%, *p* = 0.91) readmission rate and results were not altered by propensity score matching. **Conclusions**: Aprepitant use as an abortive therapy did not affect the 7-day CVS-related readmission rate. Further prospective studies are needed to explore the role of aprepitant as an abortive agent in the management of CVS in the pediatric population.

## 1. Introduction

Cyclic vomiting syndrome (CVS) is a disorder of gut–brain interactions (DGBI) characterized by repeated, stereotypical episodes of nausea and vomiting with a return to baseline health in between these episodes [1]. Consequently, patients undergo great distress from these symptoms, including repeated hospitalizations for fluid replacement and symptom management resulting in missed school/workdays, increased health care expenditure, and poor quality of life [2,3,4,5,6]. They also have increased association with disorders such as migraine, other DGBI, and psychiatric comorbidities [3,5,7]. In CVS, the mainstay of abortive therapy includes administration of antiemetics and fluid management. Ondansetron is the most commonly used abortive agent and sumatriptans and aprepitant are increasingly utilized as second line agents [8,9].

Aprepitant, a neurokinin-1 receptor antagonist, exerts its action by preventing the binding of substance P to the neurokinin-1 receptor. Neurokinin-1 receptors are present in the area postrema and solitary nucleus and aprepitant targets the central brain pathways that signal the vomiting reflex and symptoms of nausea and concomitant abdominal pain. The safety and efficacy of aprepitant has been well studied in many disorders such as chemotherapy-induced vomiting and postoperative vomiting [10,11,12]. In adults, aprepitant has also been increasingly utilized in the management of intractable nausea and vomiting related to CVS and gastroparesis [13,14,15]. However, there is limited data on the impact of aprepitant on the readmission of CVS patients in the pediatric age group.

The objective of this study was to analyze the efficacy of aprepitant on the readmission for CVS in the pediatric age group. We hypothesized that aprepitant use may be associated with a decrease in the 7-day readmission rate. We also hypothesized that aprepitant use may be associated with severe CVS.

## 2. Methods

We analyzed the Pediatric Health Information System (PHIS) database which is an aggregation of inpatient, observation, emergency department, and ambulatory surgery encounter data from more than 50 children’s hospitals across the United States affiliated with the Children’s Hospital Association (Lenexa, KS) [16]. This database was created for the purposes of external benchmarking and quality improvement, the validity and reliability of the datasets are ensured both by the Children’s Hospital Association and the partnering hospitals [17]. The dataset includes patient demographics, admission details, comorbid diagnoses, and procedures based on the International Classification of Diseases, 9th Revision and 10th Revision, Clinical Modification (ICD-9/10-CM) and Current Procedural Terminology codes [17]. Hospitals also submit resource utilization data based on clinical, pharmaceutical, laboratory, and imaging charges. In the PHIS database, data are deidentified at submission and are subjected to reliability and validity checks before inclusion [17].

We included all patients less than 18 years of age admitted to the hospital inpatient or observation units with a primary diagnosis of CVS. We analyzed all patients with hospitalization between 2016 and 2019 during which the International Classification of Diseases (ICD) 10th Revision (ICD-10) codes were employed for coding and billing purposes, thus having a specific code for CVS which was not available in the previous version of ICD. CVS patients who received aprepitant (referred to as the aprepitant group) during their index inpatient hospitalization for CVS were compared with CVS inpatients who were treated without aprepitant (referred to as the control group).

We analyzed various demographic variables, comorbid medical and psychiatric conditions, and management details during their hospital stay. In PHIS, participating hospitals submit race and ethnicity data for each visit according to hospital-specific practices, which include parent/guardian self-report at the time of arrival or hospital registration assignment. The comorbid medical and psychiatric conditions analyzed were migraine, gastroesophageal reflux disease (GERD), anxiety, cannabis use, depression, abdominal migraine, dyspepsia, dysautonomia, obesity, narcotic use, alcohol use, smoking, adjustment disorders, stress reaction, post-traumatic disorders (PTSD), and bipolar disorders as these conditions are increasingly prevalent among patients with CVS and may potentially affect readmission rates after hospitalization [5,18].

The primary outcome variable was the readmission rate within 7 days of the index hospitalization. Secondary outcome variables analyzed were the length of stay, total hospitalization expense of the index hospitalization, and readmission rates at 14 days, 30 days, 90 days, 180 days, and 365 days. As the timing of aprepitant use during the hospitalization could not be obtained in this retrospective study, these secondary outcome variables were not used to determine the efficacy of aprepitant but rather used to analyze the severity of the CVS.

### Statistical Analysis

All continuous variables were expressed as median with interquartile range and categorical variables were expressed as frequencies and percentages. All categorical variables were compared using the chi-square test of independence and Fisher’s exact test as applicable and continuous variables were compared using Wilcoxon rank-sum test. The readmission rates were calculated between the study groups and then recalculated after 1:5 propensity score matching (PSM). PSM was performed using MatchIt package in R Studio with the following demographics and clinical comorbidities: covariates: race, ethnicity, insurance, severity level of the disease, and the number of drugs prescribed. A *p* value < 0.05 was considered statistically significant. All the analyses were performed using SAS software, Version 9.4 (SAS Institute, Cary, NC, USA) and R software, Version 4.3.1.

## 3. Results

A total of 1775 pediatric patients were analyzed who were hospitalized with a primary diagnosis of CVS during the study period between 2016 and 2019. A total of 96 patients received aprepitant during the index hospitalization and 1679 patients with CVS did not receive aprepitant during their index stay. Patients who received aprepitant were slightly older, more often females, and had private insurance, but these differences were not statistically significant (Table 1). Patients in the aprepitant group significantly belonged to the Caucasian race and non-Hispanic ethnicity, the aprepitant group had slightly lower family income and higher disease severity (as coded by the clinician using the ICD-10). However, no statistical differences were noted between the cohorts for these variables.

The aprepitant group had an increased prevalence of anxiety, depression, abdominal migraine, dysautonomia, PTSD, and bipolar disorder but the differences were not statistically significant. The prevalence of GERD, cannabis use, dyspepsia, obesity, smoking, narcotic use, alcohol use, stress reaction, and adjustment disorder were decreased (or absent) in the aprepitant group and the differences were not statistically significant.

The length of stay, hospitalization charges, and costs were significantly higher in the aprepitant group (*p* < 0.001 for all these variables, (Table 1)). The readmission rates within 7 days, 14 days, and 30 days of index hospitalization were slightly high in the aprepitant group but no statistical differences were noted. However, patients receiving aprepitant had significantly greater readmission rates at 90 days (70% vs. 57%, *p* = 0.020), 180 days (81% vs. 68%, *p* = 0.010), and 365 days (85% vs. 76, *p* = 0.040 (Supplemental Table S1)).

After 1:5 PSM, there were 96 in the aprepitant group and 480 in the control group with a total of 576 patients (Table 2). All the variables in demographics, and comorbid conditions remain statistically not significant between the groups after PSM. We also compared the medication use between the groups which was overall very similar except for promethazine and diphenhydramine. Promethazine use was significantly lower in the aprepitant group (18% vs. 28%, *p* = 0.043) and conversely, diphenhydramine use was significantly higher in the aprepitant group (76% vs. 64%, *p* = 0.03). The length of stay (5.0 vs. 3.0 days, *p* < 0.001), hospital charges ($31,764 vs. $22,207, *p* < 0.001), and hospital costs ($11,790 vs. $6690, *p* < 0.001) remained significantly increased for the aprepitant group (Table 2).

In assessing readmission rates after PSM, there was no significant difference in the 7-day (17% vs. 16%, *p* = 0.91), 14-day (29% vs. 28%, *p* = 0.83), or 30-day (47% vs. 41%, *p* = 0.38) readmission rates. Patients in the aprepitant group had slightly higher readmission rates at 90 days (70% vs. 60%, *p* = 0.09), 180 days (81% vs. 72%, *p* = 0.07), and 365 days (85% vs. 79%, *p* = 0.16) but did not reach statistical significance (Supplemental Table S2).

## 4. Discussion

Using national-level data, we demonstrated that aprepitant use during the index hospital stay did not result in a reduction of 7-day readmission rate. The aprepitant group had an increased duration of hospital stay and total hospitalization expenses. There was no difference in readmission rates between the groups at 14- and 30 days. Further, no significant differences were noted in the readmission rates between the groups at 90, 180, and 365 days after the PSM.

CVS guidelines in adults published in 2019 emphasized the adjunct role of aprepitant for both prevention as well for acute management [18]. In adults with CVS, Patel and colleagues demonstrated that prophylactic use of aprepitant did significantly decrease the total number of CVS episodes from 14.5 to 6.2 and also decreased hospitalizations from 1.6 to 0.8, *p* = 0.02 [14]. The North American Society for Pediatric Gastroenterology, Hepatology, and Nutrition (NASPGHAN) consensus statement on CVS was published in 2008 and recommended the use of 5HT_3_ receptor antagonists (such as ondansetron or Granisetron) or 5HT_1B_/_1D_ agonists (such as sumatriptan) for abortive management. However, in recent years, the use of aprepitant for managing severe CVS has been increasing based on expert recommendations [19,20,21,22,23]. A single-center study involving a smaller sample in the pediatric population also supported both the acute and prophylactic use of aprepitant in CVS. Aprepitant use was associated with significant reduction in several parameters such as annual CVS episodes, hospital admissions/year, severity of CVS, and improvement in symptom-free interval duration and better school attendance [24]. Here, the authors noted that in 25 pediatric patients with CVS refractory to conventional medications, the use of aprepitant during the early prodromal phase resulted in symptomatic improvement in approximately three-fourths of the patients [24].

In our study, aprepitant use during the index hospital stay did not result in the reduction in 7-day readmission rate. Various reasons could be explained for this lack of significance between the groups. Given limited data on the utility of aprepitant in the pediatric population, we believe that aprepitant is used preferentially as second or third-line medication for severe CVS phenotype. In support of this concept, the aprepitant group had a significantly more severe hospitalization course as noted by an increased duration of hospital stay and total hospitalization expenses. Given the methodology utilized, we were not able to determine the important details of aprepitant therapy such as the timing of administration, frequency and duration of use, dosage details, and response to therapy which might have provided more information on this efficacy.

There are several other limitations which should be taken into account. Since ICD coding was used to assess for cases of CVS, any errors or variances in coding practices may have excluded patients from the study or affected how data were recorded and interpreted. -In this study, patient information was gathered mostly from tertiary care hospitals where there is ready access to specialists and more advanced treatments. Thus, referral bias may have been introduced as these facilities would be seeing a more severe spectrum of CVS cases compared to community hospitals. Specific details relating to the severity and course of the disease were unavailable, such as data related to the number of episodes of emesis, dose and clinical response to medications, details of intravenous hydration, and the time between onset of symptoms and hospital admission. Any medical care received outside of the hospital admission was also unavailable in the database, further limiting the assessment of the CVS disease course.

Despite these limitations, our study has numerous strengths as well. We had a large national-level sample size and analyzed readmission rates at various intervals. Data were taken from when ICD-10 was largely implemented in medical practice, thus minimizing the issue of coding errors and variances previously discussed. Multiple, frequent quality checks were also performed at different points of data collection to ensure reliability of the data through the PHIS database. Further, we used PSM utilizing a nearest matching method with a ratio of 1:5 which helped reducing the confounding variables between the groups.

## 5. Conclusions

Our study demonstrated that patients in the aprepitant group had a prolonged hospitalization course. Aprepitant use during index hospital stay did not reduce readmission rate in both short- and long-term intervals. Further prospectives are required to confirm or refute our results.

## Figures and Tables

**Table 1:** Comparison of baseline demographics, clinical and outcome characteristics between the population.

Variables	Overall, N = 1775 ^1^	Aprepitant Group, N = 96 ^1^	No Aprepitant Use, N = 1679 ^1^	*p*-Value ^2^
**Age (years)**	12.0 (8.0, 16.0)	13.5 (6.0, 16.0)	12.0 (8.0, 16.0)	0.426
**Gender**				0.130
Female	1081 (61%)	66 (69%)	1015 (60%)	
Male	694 (39%)	30 (31%)	664 (40%)	
**Race**				**0.014**
Black	346 (19%)	15 (16%)	331 (20%)	
Other	327 (18%)	10 (10%)	317 (19%)	
Unknown	47 (2.6%)	0 (0%)	47 (2.8%)	
White	1055 (59%)	71 (74%)	984 (59%)	
**Ethnicity**				**0.006**
Hispanic or Latino	300 (17%)	11 (11%)	289 (17%)	
Not Hispanic or Latino	1384 (78%)	85 (89%)	1299 (77%)	
Unknown	91 (5.1%)	0 (0%)	91 (5.4%)	
**Insurance**				0.060
Private	808 (46%)	57 (59%)	751 (45%)	
Public	813 (46%)	36 (38%)	777 (46%)	
Uninsured	17 (1.0%)	0 (0%)	17 (1.0%)	
Others	134 (7.5%)	3 (3.1%)	131 (7.8%)	
**Income**				
Known income ($)	44,371 (35,791, 59,505)	41,410 (38,516, 64,816)	44,371 (35,693, 59,350)	0.953
Unknown income (n)	72	3	69	
**Severity Level**				0.060
Extreme	29 (1.6%)	2 (2.1%)	27 (1.6%)	
Major	439 (25%)	23 (24%)	416 (25%)	
Moderate	828 (47%)	55 (57%)	773 (46%)	
Minor	479 (27%)	16 (17%)	463 (28%)	
**Comorbid Conditions**				
Migraine	1775 (100%)	96 (100%)	1679 (100%)	
GERD	369 (21%)	12 (12%)	357 (21%)	0.054
Anxiety	379 (21%)	28 (29%)	351 (21%)	0.073
Cannabis use	189 (11%)	5 (5.2%)	184 (11%)	0.108
Depression	116 (6.5%)	7 (7.3%)	109 (6.5%)	0.923
Abdominal migraine	94 (5.3%)	9 (9.4%)	85 (5.1%)	0.109
Dyspepsia	69 (3.9%)	2 (2.1%)	67 (4.0%)	0.583
Dysautonomia	27 (1.5%)	4 (4.2%)	23 (1.4%)	0.054
Obesity	32 (1.8%)	0 (0%)	32 (1.9%)	0.414
Narcotic use	4 (0.2%)	0 (0%)	4 (0.2%)	>0.999
Alcohol use	1 (<0.1%)	0 (0%)	1 (<0.1%)	>0.999
Smoking	17 (1.0%)	0 (0%)	17 (1.0%)	>0.999
Adjustment Disorder	41 (2.3%)	2 (2.1%)	39 (2.3%)	>0.999
Stress reaction	3 (0.2%)	0 (0%)	3 (0.2%)	>0.999
PTSD	28 (1.6%)	2 (2.1%)	26 (1.5%)	0.661
Bipolar disorder	21 (1.2%)	2 (2.1%)	19 (1.1%)	0.316
**Length of stay (days)**	3.00 (2.00, 5.00)	5.00 (3.00, 8.00)	3.00 (2.00, 5.00)	**<0.001**
**Hospital charges ($)**	21,496 (13,444, 35,410)	31,764 (19,316, 61,943)	21,054 (13,304, 34,792)	**<0.001**
**Hospital costs ($)**	6560 (4092, 10,696)	11,790 (6879, 20,767)	6380 (4000, 10,270)	**<0.001**
**Readmission < 7 Days**				0.918
No	1495 (84%)	80 (83%)	1415 (84%)	
Yes	280 (16%)	16 (17%)	264 (16%)	
**Readmission < 14 Days**				0.299
No	1345 (76%)	68 (71%)	1277 (76%)	
Yes	430 (24%)	28 (29%)	402 (24%)	
**Readmission < 30 days**				0.103
No	1092 (62%)	51 (53%)	1041 (62%)	
Yes	683 (38%)	45 (47%)	638 (38%)	

(GERD—gastroesophageal reflux disease, PTSD—post-traumatic stress disorder). ^1^ Statistics presented: Median (IQR); n (%). ^2^ Statistical tests performed: Wilcoxon rank-sum test; Fisher’s exact test; chi-square test of independence as appropriate.

**Table 2:** Comparison of various demographics, clinical and outcome characteristics after propensity score matching.

Variables	Overall, N = 576 ^1^	Aprepitant, N = 96 ^1^	No Aprepitant, N = 480 ^1^	*p*-Value ^2^
**Age (years)**	13.0 (9.0, 16.0)	13.5 (6.0, 16.0)	13.0 (9.0, 16.0)	0.129
**Gender**				0.204
Female	360 (62%)	66 (69%)	294 (61%)	
Male	216 (38%)	30 (31%)	186 (39%)	
**Race**				0.786
Black	104 (18%)	15 (16%)	89 (19%)	
Other	56 (9.7%)	10 (10%)	46 (9.6%)	
White	416 (72%)	71 (74%)	345 (72%)	
**Ethnicity**				0.855
Hispanic or Latino	60 (10%)	11 (11%)	49 (10%)	
Not Hispanic or Latino	516 (90%)	85 (89%)	431 (90%)	
**Severity level**				0.962
Extreme	13 (2.3%)	2 (2.1%)	11 (2.3%)	
Major	151 (26%)	23 (24%)	128 (27%)	
Minor	93 (16%)	16 (17%)	77 (16%)	
Moderate	319 (55%)	55 (57%)	264 (55%)	
**Insurance**				0.464
Others	11 (1.9%)	3 (3.1%)	8 (1.7%)	
Private	328 (57%)	57 (59%)	271 (56%)	
Public	237 (41%)	36 (38%)	201 (42%)	
**Income**				
Known income ($)	46,913 (37,754, 64,592)	41,410 (38,516, 64,816)	47,755 (37,710, 64,346)	0.137
Unknown income (n)	22	3	19	
**Comorbid conditions**				
Migraine	576 (100%)	96 (100%)	480 (100%)	
GERD	108 (19%)	12 (12%)	96 (20%)	0.115
Anxiety	148 (26%)	28 (29%)	120 (25%)	0.468
Cannabis use	58 (10%)	5 (5.2%)	53 (11%)	0.122
Depression	41 (7.1%)	7 (7.3%)	34 (7.1%)	>0.999
Abdominal migraine	40 (6.9%)	9 (9.4%)	31 (6.5%)	0.420
Dyspepsia	21 (3.6%)	2 (2.1%)	19 (4.0%)	0.553
Dysautonomia	11 (1.9%)	4 (4.2%)	7 (1.5%)	0.094
Obesity	7 (1.2%)	0 (0%)	7 (1.5%)	0.607
Narcotic use	2 (0.3%)	0 (0%)	2 (0.4%)	>0.999
Alcohol use	0 (0%)	0 (0%)	0 (0%)	
Smoking	4 (0.7%)	0 (0%)	4 (0.8%)	>0.999
Adjustment disorder	12 (2.1%)	2 (2.1%)	10 (2.1%)	>0.999
Stress reaction	0 (0%)	0 (0%)	0 (0%)	
PTSD	14 (2.4%)	2 (2.1%)	12 (2.5%)	>0.999
Bipolar disorder	10 (1.7%)	2 (2.1%)	8 (1.7%)	0.676
**Use of other medications**				
Ondansetron	504 (88%)	80 (83%)	424 (88%)	0.237
Lorazepam	364 (63%)	62 (65%)	302 (63%)	0.847
Sumatriptan	20 (3.5%)	4 (4.2%)	16 (3.3%)	0.758
Promethazine	153 (27%)	17 (18%)	136 (28%)	**0.043**
Granisetron	34 (5.9%)	6 (6.2%)	28 (5.8%)	>0.999
Diphenhydramine	381 (66%)	73 (76%)	308 (64%)	**0.033**
**Length of stay (days)**	3.0 (2.0, 5.0)	5.0 (3.0, 8.0)	3.0 (2.0, 5.0)	**<0.001**
**Hospital charges ($)**	23,428 (14,914, 38,975)	31,764 (19,316, 61,943)	22,207 (14,688, 37,108)	**<0.001**
**Hospital costs ($)**	7048 (4716, 12,380)	11,790 (6879, 20,767)	6690 (4332, 10,965)	**<0.001**
**Readmission < 7 days**				>0.999
No	479 (83%)	80 (83%)	399 (83%)	
Yes	97 (17%)	16 (17%)	81 (17%)	
**Readmission < 14 days**				
No	416 (72%)	68 (71%)	348 (72%)	0.835
Yes	160 (28%)	28 (29%)	132 (28%)	
**Readmission < 30 days**				0.386
No	332 (58%)	51 (53%)	281 (59%)	
Yes	244 (42%)	45 (47%)	199 (41%)	

(GERD—gastroesophageal reflux disease, PTSD—post-traumatic stress disorder). ^1^ Statistics presented: Median (IQR); n (%). ^2^ Statistical tests performed: Wilcoxon rank-sum test; Fisher’s exact test; chi-square test of independence as appropriate.

## Data Availability

The data presented in this study are available at the Pediatric Health Information System (PHIS) database at https://www.childrenshospitals.org/content/analytics/product-program/pediatric-health-information-system (accessed on 10 January 2021).

## Supplementary Materials

The following supporting information can be downloaded at: https://www.mdpi.com/article/10.3390/medicines11080021/s1, Table S1: Comparison between the groups before the propensity matching; Table S2: Comparison between the groups after propensity matching.
