# Supplementary material for: Utility of Aprepitant in the Management of Pediatric Patients with Cyclical Vomiting Syndrome
Source: Medicines (Basel). 2024 Dec 11;11(8):21. doi: 10.3390/medicines11080021 (PMC11677684; doi:10.3390/medicines11080021)
Supplement: Supplementary file 1 [file medicines-11-00021-s001.zip › medicines-3157031-supplementary.pdf]

Supplementary Table S1 – The readmission rates between the groups (before the propensity matching)

Variables	Total patients N = 1,775[1]	Aprepitant, N = 96[1]	No Aprepitant, N = 1,679[1]	p-value[2]
Readmission <90 Days				0.020
No	748 (42%)	29 (30%)	719 (43%)	
Yes	1,027 (58%)	67 (70%)	960 (57%)	
Readmission <180 Days				0.010
No	551 (31%)	18 (19%)	533 (32%)	
Yes	1,224 (69%)	78 (81%)	1,146 (68%)	
Readmission <365 Days				0.040
No	422 (24%)	14 (15%)	408 (24%)	
Yes	1,353 (76%)	82 (85%)	1,271 (76%)	

[1]Statistics presented: Median (IQR); n (%)
[2]Statistical tests performed: Wilcoxon rank-sum test; chi-square test of independence; Fisher's exact test

Supplementary Table S2 - The readmission rates between the groups (after the propensity matching)

Variables	Overall, N = 576[1]	Aprepitant, N = 96[1]	No Aprepitant, N = 480[1]	p-value[2]
Readmission <90 Days				0.099
No	220 (38%)	29 (30%)	191 (40%)	
Yes	356 (62%)	67 (70%)	289 (60%)	
Readmission <180 Days				0.076
No	153 (27%)	18 (19%)	135 (28%)	
Yes	423 (73%)	78 (81%)	345 (72%)	
Readmission <360 Days				0.165
No	117 (20%)	14 (15%)	103 (21%)	
Yes	459 (80%)	82 (85%)	377 (79%)	

[1]Statistics presented: Median (IQR); n (%)
[2]Statistical tests performed: Wilcoxon rank-sum test; chi-square test of independence; Fisher's exact test
